# Early cardiac lipid overload, redox-inflammatory imbalance, fibrotic remodeling, and cannabinoid receptor up-regulation in sucrose-rich diet-fed rats: preventive effects of full-spectrum cannabis oil

**DOI:** 10.3389/fnut.2026.1945796

**Published:** 2026-10-30

**Authors:** Valentina Degrave, Paola Ingaramo, Laura Caltana, Hugo H. Ortega, Facundo J. Salinas, Daniela Sedan, Darío Andrinolo, María Eugenia D’Alessandro, María Eugenia Oliva

**Affiliations:** 1 Laboratorio de Estudio de Enfermedades Metabólicas relacionadas con la Nutrición, Facultad de Bioquímica y Ciencias Biológicas, Universidad Nacional del Litoral, Santa Fe, Argentina; 2 Instituto de Salud y Ambiente del Litoral (ISAL), Facultad de Bioquímica y Ciencias Biológicas, Consejo Nacional de Investigaciones Científicas y Técnicas (CONICET), Santa Fe, Argentina; 3 Instituto de Biología Celular y Neurociencias Prof E De Robertis, Facultad de Medicina, Universidad de Buenos Aires, Buenos Aires, Argentina; 4 Centro de Medicina Comparada, Instituto de Ciencias Veterinarias del Litoral (ICiVet-Litoral), Universidad Nacional del Litoral (UNL), Consejo Nacional de Investigaciones Científicas y Técnicas (CONICET), Esperanza, Santa Fe, Argentina; 5 Centro de Investigaciones del Medio Ambiente (CIM)/Consejo Nacional de Investigaciones Científicas y Técnicas (CONICET) –Universidad Nacional de La Plata, La Plata, Argentina; 6 Consejo Nacional de Investigaciones Científicas y Técnicas (CONICET), Santa Fe, Argentina

**Keywords:** bioactive compounds, cannabinoid receptors, cardiac metabolic remodeling, full-spectrum cannabis oil, inflammation, lipid metabolism, oxidative stress, sucrose-rich diet

## Abstract

**Introduction:**

Excessive intake of added sugars promotes metabolic disease, but the earliest myocardial responses to sugar-driven metabolic overload and their modulation by plant-derived bioactive compounds remain incompletely understood. We investigated whether a short-term sucrose-rich diet induces early cardiac remodeling and whether a full-spectrum cannabis oil (CO) can prevent these alterations.

**Methods:**

Male Wistar rats were assigned for 3 weeks to a reference diet, a sucrose-rich diet, or a sucrose-rich diet plus full-spectrum CO (cannabidiol [CBD]: tetrahydrocannabinol [THC] ratio 2:1; 1 mg/kg/day, orally).

**Results:**

Throughout the study period, the sucrose-rich diet significantly increased systolic and diastolic blood pressure (group effect, *p* = 0.0003 and *p* = 0.0029, respectively) and circulating cardiac injury markers (creatine kinase-MB [CK-MB], *p* = 0.0048; lactate dehydrogenase [LDH], *p* = 0.0173), promoted myocardial triglyceride accumulation (*p* = 0.0002), and altered lipid metabolism-related regulators (e.g., fatty acid synthase [FAS], *p* = 0.0002; sterol regulatory element-binding protein 1c [SREBP-1c], *p* = 0.0007). These changes were accompanied by oxidative stress (reactive oxygen species [ROS], *p* < 0.0001), impaired antioxidant defenses (catalase [CAT], *p* < 0.0001), inflammatory and endothelial activation (nuclear factor-κB [NF-κB], *p* = 0.0049; vascular cell adhesion molecule-1 [VCAM-1], *p* < 0.0001), early fibrotic remodeling (myocardial collagen content, *p* < 0.0001), histological and ultrastructural injury, and increased cardiac cannabinoid receptor type 1 (CB_1_R, *p* = 0.0001) and cannabinoid receptor type 2 (CB_2_R, *p* = 0.0003) protein expression, whereas electrocardiographic parameters (heart rate, PR interval, QRS complex, corrected QT interval [QTc]) remained unchanged (all *p* > 0.05).

**Discussion:**

Cannabis oil administration prevented or markedly attenuated most of these alterations, including cardiac lipid accumulation, redox imbalance, inflammatory and remodeling-related signaling, structural damage, and cannabinoid receptor upregulation. These findings show that the myocardium is an early target of sucrose-driven metabolic overload and identify full-spectrum CO as a plant-derived bioactive intervention capable of limiting early cardiac metabolic and redox-inflammatory remodeling in this preclinical model. Further studies are required to define the contribution of individual phytocannabinoids and to determine whether cannabinoid receptor signaling contributes to these effects.

## Graphical abstract

**Figure:**
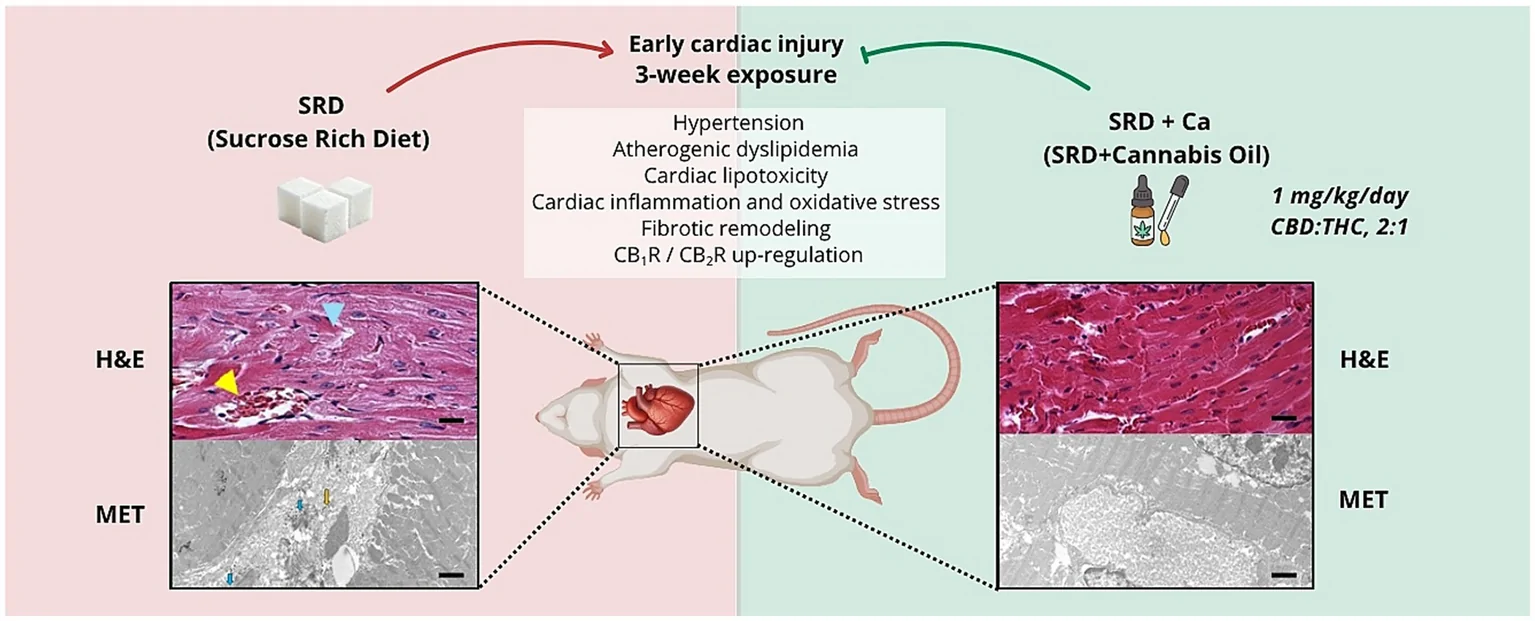

## 1 Introduction

Over the past decades, dietary patterns have shifted toward a sustained overconsumption of free sugars, particularly through sucrose-rich processed foods, often exceeding the intake limits recommended by the World Health Organization (1, 2). This nutritional environment has been consistently associated with disrupted lipid metabolism, oxidative stress, and low-grade inflammation, which collectively contribute to early cardiometabolic dysfunction and increased cardiovascular risk (3, 4). Although metabolic syndrome (MetS) is commonly used to conceptualize this set of alterations, they may also reflect a state of sugar-induced metabolic overload capable of altering hepatic lipid metabolism (5) and promoting a pro-oxidant and pro-inflammatory systemic environment (6). This context is clinically relevant, given that MASLD is closely associated with increased cardiovascular risk through shared cardiometabolic, inflammatory, oxidative, and endothelial mechanisms (7). In this framework, metabolic and inflammatory crosstalk between organs can extend the consequences of sugar overload beyond the liver, contributing to early cardiac alterations described after high exposures to fructose (8, 9) or sucrose (10, 11).

The myocardium is particularly vulnerable to metabolic imbalance because cardiac performance is tightly coupled to substrate availability, uptake, and oxidation. Under physiological conditions, the heart preserves metabolic flexibility by adapting substrate utilization to energetic demand (12, 13). However, under cardiometabolic stress, this flexibility may be compromised, leading to increased lipid influx, altered substrate oxidation, and cardiac triglyceride accumulation, a hallmark of cardiac lipid overload (14–17). When lipid supply exceeds oxidative capacity, toxic lipid intermediates, mitochondrial dysfunction and excessive reactive oxygen species (ROS) production, disrupts cardiac bioenergetics and promotes inflammatory activation (18–21).

Beyond bioenergetic impairment, sustained lipotoxic, oxidative, and inflammatory stress converges on the cardiac extracellular matrix where pro-oxidant and pro-inflammatory signaling promotes fibroblast activation and extracellular matrix deposition, driving interstitial and perivascular fibrosis. This process impairs myocardial elasticity and represents an early structural component of metabolic cardiac remodeling (14, 19, 22). Because pro-fibrotic signaling can be engaged before overt contractile or electrical dysfunction becomes apparent, its activation may constitute one of the earliest detectable myocardial responses to sugar overload (23, 24).

In this context, sucrose-rich diet (SRD) models provide a useful experimental approach to investigate diet-induced cardiac alterations, as they reproduce several cardiometabolic features observed in human metabolic disorders (25, 26). Previous studies from our group have demonstrated that chronic SRD feeding to rats induces hypertension, cardiac lipid accumulation, oxidative stress, inflammatory activation, and fibrotic remodeling (27–29). However, whether short-term exposure to SRD is sufficient to trigger an integrated molecular, biochemical, and structural cardiac response —including early fibrotic signaling— before overt electrical dysfunction becomes evident remains unclear. Among the molecular pathways potentially involved in this response, the endocannabinoid system (ECS) has emerged as an important regulator of metabolic, redox, inflammatory, and cardiovascular homeostasis, integrating nutrient sensing with cellular stress responses. Cannabinoid receptor type 1 (CB_1_R) and type 2 (CB_2_R) are expressed in the myocardium and participate in the regulation of substrate metabolism, vascular tone, immune responses, and cellular adaptation to stress (30–32). In experimental settings, CB_1_R activation has been associated with increased lipogenesis, oxidative stress, and pro-inflammatory responses, whereas CB_2_R activation has been linked to immunomodulatory and potentially protective and antifibrotic effects (31, 33, 34). Therefore, ECS dysregulation may contribute to the myocardial response to metabolic overload, including redox-inflammatory imbalance and extracellular matrix remodeling (35–38). Cardiac regulation of these receptors appears to be context dependent and may vary according to the severity and inflammatory burden of tissue injury. Increased myocardial CB_1_R expression has been reported in diet-induced and genetic models of obesity, as well as in human cardiometabolic disease (39–41). By contrast, CB_2_R expression remained unchanged in some diet-induced or obesity models (40, 41), whereas increased cardiac CB_2_R expression has been described in diabetic cardiomyopathy and advanced heart failure, conditions characterized by a more pronounced inflammatory component (32, 33). In these settings, CB_2_R upregulation has been proposed to reflect an adaptive or counter-regulatory response to inflammatory stress. However, receptor abundance does not necessarily indicate functional activation, and the biological consequences of CB_2_R signaling may vary according to cell type, disease stage, and pharmacological context (42). Whether an early nutritional challenge driven exclusively by sucrose is sufficient to modify myocardial CB_2_R expression therefore remains unclear.

In parallel, exogenous cannabinoids—particularly phytocannabinoids such as cannabidiol (CBD) (43–45) and Δ9-tetrahydrocannabinol (THC) (34, 46) —modulate redox balance (47), mitochondrial function, and inflammatory pathways. Consistent with these actions, cannabinoid-based interventions have shown vasodilatory, antioxidative, anti-inflammatory, and cardioprotective effects across different cardiovascular settings. Importantly, the cardiometabolic actions of cannabis-based preparations cannot be inferred from the receptor-level pharmacology of individual cannabinoids. In full-spectrum extracts, phytocannabinoids, terpenes, and minor constituents act in combination, generating a net biological response that reflects synergistic and modulatory interactions, commonly referred to as the ‘entourage effect’ (48). Accordingly, the present study was not designed to dissect the contribution of individual constituents, but rather to evaluate the integrated effects of a defined orally administered full-spectrum CO (CBD:THC, 2:1), an approach with greater translational relevance.

Building on our previous work showing that this full-spectrum CO (CBD:THC, 2:1) modulates hepatic and systemic metabolic alterations in the SRD model (49, 50), the present study extends this line of research to the cardiac tissue, an organ not previously examined under this intervention. To our knowledge, however, no study has evaluated whether a full-spectrum oil can prevent the earliest stages of cardiac remodeling in a diet-induced metabolic model before overt disease becomes established.

Therefore, the present study aimed to determine whether short-term SRD feeding is sufficient to induce early cardiac alterations, including myocardial lipid accumulation, redox-inflammatory imbalance, fibrotic signaling, and changes in cannabinoid receptor expression, and to evaluate whether oral co-administration of a full-spectrum CO (CBD:THC, 2:1) from the onset of dietary exposure prevents these alterations.

## 2 Materials and methods

### 2.1 Cannabis oil preparation and characterization

The CO was obtained from dried and mature inflorescences of the *Cannabis sativa* variety CAT1, grown at the Environmental Research Center (CIM-CONICET-UNLP) (RESOL-2021-3236-APN-MS), as previously described by Degrave et al. (50). The dried and ground inflorescences were decarboxylated and subsequently subjected to ethanolic extraction. Ethanol was removed by rotary evaporation, and the resulting resin was diluted in corn oil, which was also used as the vehicle in the control groups, to obtain a working concentration of 1 mg/mL.

The phytocannabinoid profile of the batch used was determined by high-performance liquid chromatography with diode array detector (HPLC-DAD) at the Drug Quality Control Laboratory, FBCB-UNL. The oil contained CBD (0.68 mg/mL), CBDA (0.34 mg/mL), Δ9-THC (0.47 mg/mL), THCA (0.03 mg/mL), CBC (0.05 mg/mL), CBGA (0.02 mg/mL) and CBG (0.02 mg/mL). The concentrations of total CBD and total THC, considering their respective acid precursors, were 0.99 and 0.50 mg/mL, respectively, corresponding to an approximate CBD:THC ratio of 2:1. CBDVA, CBDV, THCV, THCVA, CBN and Δ8-THC were not detected. Terpene analysis identified four sesquiterpenes: (−)-α-bisabolol (41.1 ppm), (−)-guaiol (29.8 ppm), β-caryophyllene (24.1 ppm) and α-humulene (13.8 ppm), with a total terpene content of 108.8 ppm. This profile corresponds to the same formulation previously characterized by our group (51).

### 2.2 Animals and diets

Adult male Wistar rats (*n* = 18) were purchased from the Veterinary Sciences Institute of Litoral (ICIVET-Litoral) - Faculty of Veterinary Sciences, National University of Litoral (Esperanza, Santa Fe, Argentina) and were maintained with unrestricted access to water and food under controlled temperature (22 ± 1 °C), humidity, and air flow conditions, with a fixed 12-h light/dark cycle (light on 07.00 a.m. to 7.00 p.m.). Animals were acclimated to the facility for two weeks after arrival before the onset of the dietary protocol. Adequate measures were taken to minimize pain or discomfort in the rats, and the smallest number of animals possible was used. This study was performed in strict accordance with the NIH guidelines for the care and use of laboratory animals and approved by the Institutional Ethics Committee of the Faculty of Biochemistry and Biological Sciences (UNL, Santa Fe, Argentina - Acta 03/21).

The animals were initially fed a standard powdered commercial rodent diet (GEPSA FEED, Buenos Aires, Argentine). Rats weighing 180–190 g (young adults) were allocated to the three experimental groups (n = 6/group) using a simple lot-drawing procedure, without prior stratification or blocking for 3 weeks: (1) Rats fed a standard powdered rodent commercial diet (reference diet, RD, *n* = 6), (2) Rats fed a semisynthetic sucrose-rich diet (SRD, *n* = 6) and (3) Rats fed SRD plus orally administered CO (SRD + Ca, *n* = 6). Rats received via noninvasive oral CO (CBD:THC, 2:1 ratio – SRD + Ca), or vehicle control (corn oil- RD and SRD). Cannabis oil was administered orally at a dose of 1 mg/kg body weight/day, beginning concomitantly with SRD exposure on day 1 and continuing throughout the 3-week experimental period. The semisynthetic SRD, based on a modified AIN-93 M formulation, contained (g/kg diet): sucrose 580, vitamin-free casein 165, corn oil 105, cellulose fiber 100, mineral mix 35, methionine 3, vitamin mix 10, and choline chloride 2, providing 16.3 kJ/g. This formulation replaced the starch fraction of the standard AIN-93 M diet with sucrose, which corresponded to 58% (w/w) of the diet and, based on standard Atwater conversion factors, contributed approximately 60% of total dietary energy, with protein and fat accounting for approximately 17 and 24% of energy, respectively. The RD was a commercial powdered rodent chow (GEPSA FEED, Buenos Aires, Argentina) containing (g/kg diet): carbohydrate (derived from corn, sorghum, wheat, oats, and barley) 427, protein 180, fat 39, fiber 55, minerals and vitamins 169, and moisture 130, with a manufacturer-reported digestible energy of 12.5 kJ/g. The composition of experimental diets has been described previously by Degrave et al., 2023 (52).

At the end, the food was removed at 07.00 a.m. and experiments were performed between 07.00 and 09.00 a.m. Animals were anesthetized with intraperitoneal sodium pentobarbital (60 mg/kg body weight). Blood samples were collected from the inferior vena cava and rapidly centrifuged, and serum was either immediately assayed or stored at −20 °C until use.

The whole heart was excised, weighed, and processed according to the subsequent analyses. Samples were either snap-frozen in liquid nitrogen for biochemical and molecular determinations, fixed in 10% (v/v) buffered formalin for 24 h at room temperature and embedded in paraffin for histological and immunohistochemical analyses, or fixed in 4% (w/v) paraformaldehyde and 0.25% (v/v) glutaraldehyde for transmission electron microscopy.

The general sequence of dietary interventions and administration of CO, hemodynamic recordings, and procedures performed at the end of the protocol is summarized in Figure 1.

**Figure 1:**
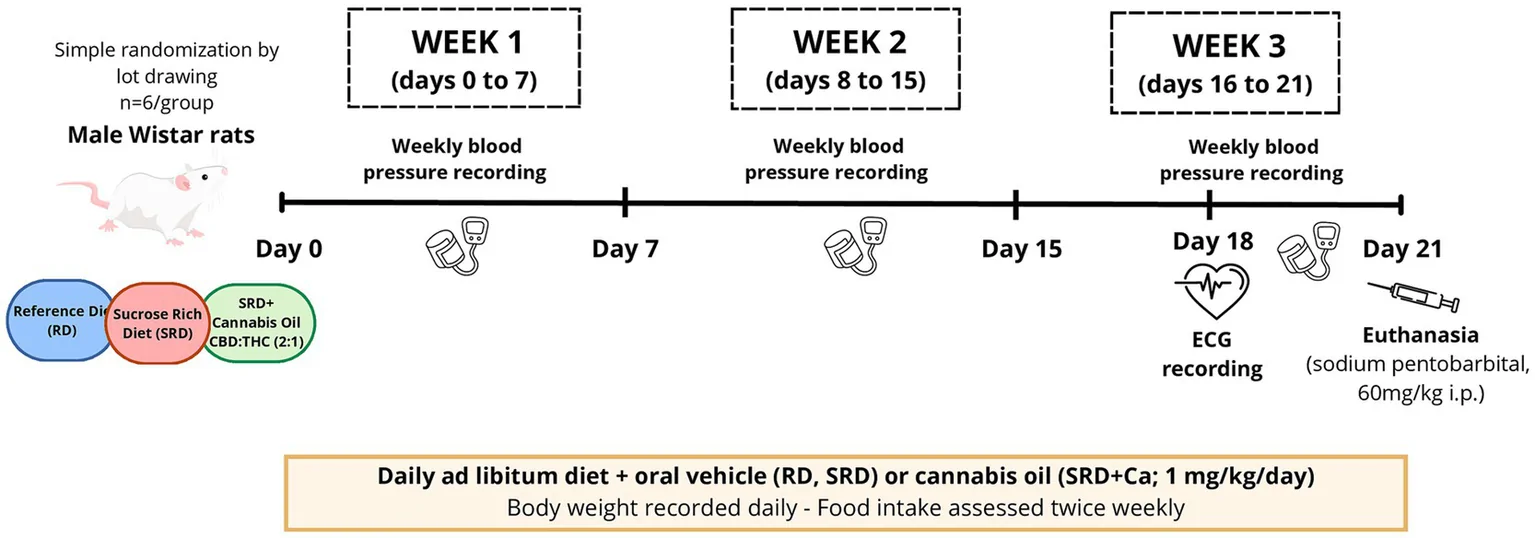
Experimental design and timeline of the study. Male Wistar rats were allocated by simple randomization using a lot-drawing procedure to three experimental groups (*n* = 6/group): reference diet (RD), sucrose-rich diet (SRD), or SRD plus full-spectrum CO (SRD + Ca; CBD:THC ratio 2:1). Animals had ad libitum access to their respective diets throughout the 3-week experimental period. The SRD + Ca group received CO orally at 1 mg/kg body weight/day from the onset of SRD exposure, whereas RD and SRD animals received the corresponding vehicle. Body weight was recorded daily and food intake was assessed twice weekly. Systolic and diastolic blood pressure were recorded weekly. Electrocardiographic (ECG) recordings were obtained on day 18. On day 21, animals were anesthetized with sodium pentobarbital (60 mg/kg body weight, i.p.) and euthanized for blood and tissue collection. CBD, cannabidiol; THC, Δ9-tetrahydrocannabinol; i.p., intraperitoneal administration.

### 2.3 Serum metabolites and enzymes, and cardiac triglyceride content

Serum glucose (Glicemia enzimática, ref. 1,400,101), triglyceride(TG Color GPO/PAP AA líquida, ref. 1,780,111), total cholesterol (Colestat enzimático, ref. 1,220,101), HDL-cholesterol (HDL Cholesterol Reactivo Precipitante, ref. 1,220,103), and LDL-cholesterol (LDL Cholesterol Reactivo Precipitante, ref. 1,220,105) levels were measured by spectrophotometric methods using commercial enzymatic kits (Wiener Lab., Rosario, Argentina) according to the manufacturer’s protocols. HDL- and LDL-cholesterol were determined by precipitation-based assays rather than by calculation. Serum non-esterified fatty acids (NEFA) concentrations were determined using the Randox Non-Esterified Fatty Acids (NEFA) Assay Kit (Randox Laboratories Ltd., Crumlin, County Antrim, UK) according to the manufacturer’s instructions. Serum activities of lactate dehydrogenase (LDH-P UV AA líquida, ref. 1,521,304) and creatine kinase-MB (CK-MB NAC UV AA líquida, ref. 1,271,361) were measured using the same commercial kit system. All spectrophotometric determinations were performed using a JASCO V-760 UV–Visible spectrophotometer (JASCO Corporation, Tokyo, Japan). Cardiac triglyceride content was determined after lipid extraction with chloroform-methanol (2:1). Aliquots were evaporated and TGs were analyzed using the enzymatic methods described above, as previously reported by Oliva et al. (29)

### 2.4 Blood pressure measurement and electrocardiographic analysis

Blood pressure was measured once per week throughout the 3-week experimental period in conscious animals using a CODA™ Monitor non-invasive blood pressure system (Kent Scientific Corporation, Torrington, CT, USA), which combines the tail-cuff occlusion method with a Volume Pressure Recording (VPR) sensor to detect tail blood volume changes; this non-invasive approach shows close agreement with direct blood pressure measurement methods (53). Prior to the first measurement, animals were acclimated to the procedure for three consecutive days (~15 min/session) by placing them in the measurement restrainers, to minimize handling-related stress. For each session, conscious animals were placed in a thermally controlled restrainer designed to gently limit movement, minimize stress, and maintain body temperature; the occlusion cuff and VPR sensor were positioned on the tail to record systolic and diastolic blood pressure. Reported values represent the average of eight valid consecutive measurement cycles per animal.

Resting cardiac electrical activity was assessed by electrocardiography (ECG) in six animals per experimental group (*n* = 18). Recordings were obtained using a seven-lead veterinary electrocardiograph (Cardio Com CC7DerS-Vet). For ECG acquisition, animals were anesthetized with inhaled isoflurane, induced in a chamber at 5% using an EZ-ANESTHESIA 190F Automatic Flow Anesthesia Machine and subsequently maintained at 2% by mask for the time required to obtain the recordings. Animals were placed in a recumbent position on a padded surface, and the corresponding electrodes were positioned for ECG acquisition. To improve electrical conductivity, 96% ethanol and neutral gel were applied at the electrode contact sites. At the end of the procedure, animals were removed from the anesthetic system and returned to their home cages for recovery.

Electrocardiographic recordings were subsequently analyzed using CC7DerS-VET 1.0.4 software. Each tracing was first subjected to an overall qualitative assessment, and a representative waveform was then selected from each record for quantitative analysis. The following electrocardiographic parameters were determined: heart rate (beats/min), QRS complex amplitude (mV), P-wave amplitude (mV), P-wave duration (ms), T-wave amplitude (mV), T-wave duration (ms), PR segment (ms), ST segment (ms), ST-segment deviation (mV), RR interval (ms), PR interval (ms), QRS interval (ms), QT interval (ms), and ST interval (ms).

The QT interval was corrected for heart rate using a rat-specific normalization of Bazett’s formula, QTcn-B = QT / (RR/150)^1/2^, where QT and RR are expressed in milliseconds and 150 ms corresponds to the mean RR interval reported for rats, as validated by Kmecová and Klimas (54) for use in this species. The original, human-derived Bazett formula (QTc = QT/√RR) was not applied, as it is calibrated for the heart rate range typical of humans (60–100 bpm) and produces systematic QTc overestimation when extrapolated to the substantially faster heart rates characteristic of rats (300–450 bpm).

### 2.5 Histological analysis

For histological examination, paraffin sections (5 μm) of whole heart samples, including both atria and ventricles, were cut and stained with Hematoxylin and Eosin (H&E) or Sirius Red (Direct Red 80, Sigma-Aldrich, Argentina) in picric acid solution (picrosirius) counterstained with Harris Hematoxylin. The heart structure and collagen organization were evaluated using Olympus BH2 light microscopy (Olympus Optical Co., Ltd., Japan). In addition, the collagen birefringence of picrosirius-stained sections was quantified by polarization microscopy. For each animal, three non-consecutive sections were analyzed, evaluating at least 10 randomly selected fields per section. Imaging and quantitative analysis were performed by an investigator blinded to experimental assignment. Collagen deposition was quantified from polarized-light images using ImageJ/Fiji software (National Institutes of Health, USA). A fixed color threshold was applied to identify birefringent collagen-positive areas, and the collagen-positive area was expressed as a percentage of the total tissue area analyzed. The same threshold settings were applied to all images across experimental groups. Imaging and histological, and morphometric analyses, were performed by an investigator blinded to experimental assignment.

### 2.6 Enzymatic activity assays

Acetyl-CoA carboxylase (ACC) enzyme activity was assayed as previously described (55). The cytosolic fraction was obtained after centrifugation of the supernatant at 30,000 rpm for 1 h at 4 °C. ACC activity was measured by an NADH-linked assay using pyruvate kinase (Cat. No. P1506), lactate dehydrogenase (Cat. No. L1254), phosphoenolpyruvate (Cat. No. P7252), ATP (Cat. No. A2382), acetyl-CoA (Cat. No. A2056), malonyl-CoA (Cat. No. M4263), and NADH (Cat. No. N4505), all purchased from Sigma-Aldrich Argentina (Buenos Aires, Argentina). Fatty acid synthase (FAS) activity was assayed in the cytosolic fraction of heart tissue by measuring malonyl-CoA-dependent oxidation of nicotinamide adenine dinucleotide phosphate (NADPH) at 37 °C, as previously described (55, 56), using NADPH (Cat. No. N1630) and malonyl-CoA (Cat. No. M4263), both purchased from Sigma-Aldrich Argentina (Buenos Aires, Argentina). Heart glucose-6-phosphate dehydrogenase (G-6-PDH), malic enzyme, and carnitine palmitoyltransferase-1 (CPT-1) activities were determined as previously described by Oliva et al. (29). G-6-PDH and malic enzyme activities were measured using NADPH (Cat. No. N1630) and NADP+ (Cat. No. N0505), while CPT-1 activity was measured using L-carnitine (Cat. No. C-0283) and palmitoyl-CoA (Cat. No. P9716). All reagents were purchased from Sigma-Aldrich Argentina (Buenos Aires, Argentina). Other chemicals and reagents used in the assays were of analytical grade. Heart reduced glutathione (GSH) content was determined according to the method of Tietze et al. (57). The activities of catalase (CAT), glutathione reductase (GR), and glutathione peroxidase (GPx) were measured in heart tissue homogenates as previously described by Creus et al. (15). Reduced glutathione (GSH; Cat. No. G4251), oxidized glutathione (GSSG; Cat. No. G4501), and NADPH (Cat. No. N1630) were purchased from Sigma-Aldrich Argentina (Buenos Aires, Argentina). Other chemicals and reagents used in the assays were of analytical grade.

### 2.7 Determination of ROS levels and lipid peroxidation

For reactive oxygen species (ROS) determination, heart tissue samples were homogenized in phosphate-buffered saline (PBS) and centrifuged at 1,000 x g for 10 min at 4 °C. ROS levels were measured in the resulting supernatant following the fluorometric method described by Ferreira et al. (56). Fluorescence intensity was normalized by protein concentration and expressed relative to the RD group. Lipid peroxidation was estimated by measuring thiobarbituric acid reactive substances (TBARS) in serum and heart tissue. For cardiac TBARS determination, samples were homogenized in a 10 mM sodium phosphate buffer (pH 7.0), and the assay was performed as previously reported by Ferreira et al. (56). Results were expressed as nmol/g tissue.

### 2.8 Immunohistochemical analysis

Immunohistochemistry was performed as previously described (58, 59). Paraffin-embedded heart sections (5 μm thickness) were used to evaluate VCAM-1 and immunoreactivity of 4-HNE-modified proteins. Sections were mounted on 3-aminopropyltriethoxysilane-coated slides (Sigma-Aldrich, Argentine), and antigen retrieval was performed by microwave pretreatment. Samples were incubated overnight (14–16 h) at 4 °C in a humid chamber with the corresponding primary antibodies against VCAM-1 (mouse monoclonal, sc-13160, Santa Cruz Biotechnology; 1:50 dilution) or 4-HNE (mouse monoclonal, MAB3249, R&D systems, 1:50 dilution), followed by incubation with a biotin-conjugated anti-mouse secondary antibody (1:100 dilution, Sigma-Aldrich, Argentina) for 30 min at room temperature. Immunoreactions were visualized using the streptavidin-biotin-peroxidase method with diaminobenzidine (Sigma-Aldrich, Argentina) as chromogenic substrate. Each run included positive and negative controls. Heart sections, known to express the target antigen, were used as positive controls. Negative controls were processed under identical conditions, except that the primary antibody was omitted and replaced with antibody diluent. Protein expression was quantified by image analysis using the Image-Pro Plus 5.0.2.9 system (Media Cybernetics, Silver Spring, MD, USA). To further ensure methodological robustness, a representative subset of immunostained images was independently re-analyzed using the open-source software ImageJ/Fiji, yielding quantitative results consistent with those obtained using the original system. Immunostained images were acquired with a DPlan 40 × objective (numerical aperture 0.65; Olympus) coupled to a Spot Insight V3.5 color video camera. For each animal, three non-consecutive sections were analyzed, evaluating at least 10 randomly selected fields per section. After grayscale conversion, IOD was determined as a linear combination of mean gray intensity and the relative area occupied by positively stained cells, as previously described by Ingaramo et al. (60). Immunohistochemical analyses were performed by an investigator blinded to experimental assignment.

### 2.9 Real-time PCR

SREBP-1c, PPARα, Nrf2, NF-kB, F4/80, TNFα, MCP-1, iNOS, TGF-β, IL-10 and PAI-1 mRNA levels were determined by semiquantitative RT-PCR analysis following protocols of Ingaramo et al. (59). Total RNA was extracted from heart tissue using the Trizol reagent (TRI Reagent®, Sigma Aldrich, USA) according to the manufacturer’s instructions. RNA concentration and purity were assessed by absorbance at 260 and 280 nm using a NanoDrop™ Lite Spectrophotometer (Thermo Fisher Scientific).

Total RNA (1 μg) was reverse-transcribed into cDNA using Moloney murine leukemia virus reverse transcriptase (Promega, Madison, WI, USA), random primers (Biodynamics SRL) and deoxynucleotide triphosphate (dNTPs). Reverse transcription was carried out at 37 °C for 90 min and at 42 °C for 15 min, followed by enzyme inactivation at 80 °C for 5 min. Each cDNA sample was diluted with nuclease-free water to a final volume of 60 μL. RT-PCR amplification was performed using a StepOne system (ThermoFisher scientific, Buenos Aires, Argentina). Each reaction contained 5 μL cDNA, SsoFastTM EvaGreen® Supermix (Bio Rad), and 10 pmol of each primer (Genbiotech) in a final volume of 20 μL. Primer sequences are listed in Table 1. After an initial denaturation at 95 °C for 15 min, amplification was performed through successive cycles of 95 °C for 15 s, 60 °C for 15 s, and 72 °C for 15 s. RNA concentration and purity were assessed spectrophotometrically at 260 and 280 nm using a NanoDrop™ Lite Spectrophotometer (Thermo Fisher Scientific). To assess potential amplification from residual genomic DNA, no-reverse-transcription (no-RT) controls were processed in parallel. No-template controls (NTCs), containing nuclease-free water instead of cDNA, were also included in the amplification assays. No amplification was detected in either type of control. Relative mRNA abundance was calculated using the relative standard curve method, normalized to the housekeeping gene L19, and expressed relative to the RD group. No significant differences in L19 Ct values were observed among experimental groups.

**Table 1:** qPCR primers sequences and product size.

Name	Forward sequences	Reverse sequences	Product size (bp)
L19	AGCCTGTGACTGTCCATTCC	TGGCAGTACCCTTCCTCTTC	99
SREBP-1c	CGCTACCGTTCCTCTATCAA	TTCGCAGGGTCAGGTTCTC	166
PPARα	CGTTGTCATCACAGATTGGTGC	CCTCTCCGAGGGACTGAGAA	183
Nrf2	GTCAGCTACTCCCAGGTTGC	GGCAAGCGACTGAAATGTAGG	135
NF-kB	ATGACAGAGGCGTGTATAAG	TCTCTCTGTCTGTGAGTTGC	109
F4/80	TACCTGCACCTGCACCTTTG	AATCCCGCAATGATAGCGCA	107
TNFα	CATCAGCAGAGCCAGGA	CATTCACCAGCGTTGCC	136
TGF-β	CGGTGCTCGCTTTGTAC	ATTGCGTTGTTGCGGTC	133
MCP-1	GGTTTTTCCACACTGGGCAAC	GGTAACCTGTTGAGAGGGCAA	71
iNOS	TGGCTGTGGTCACCTATCGC	GAAGCCACTGACACTCCGCA	128
PAI-1	AGGGCTTCATGCCCCACTTCTTCA	AGTAGAGGGCATTCACCAGCACCA	192
IL-10	ACACACAATGGAAGAATCAA	CTCAGTTACCCAAAGGAAAC	74

### 2.10 Western blot analysis

Frozen heart samples were homogenized in a RIPA buffer supplemented with protease inhibitors, left on ice for 10 min, and the supernatant was collected by centrifugation at 25,000 × g for 10 min, as previously described by our group (27). The protein concentration of homogenates was determined by the Bradford method prior to loading. 50 μg of total protein per lane was loaded for separation by SDS-PAGE in a 10% gel and electrotransferred onto PVDF membranes. The membranes were probed with mouse primary monoclonal antibodies against CB_1_R and CB_2_R (mouse monoclonal antibody; sc-518035 and sc-293188, Santa Cruz Biotechnology) and then incubated with goat anti-mouse IgG conjugated to horseradish peroxidase antibody (mIgG-Fc-BP-HRP; sc-525409, Santa Cruz Biotechnology). Specific signals were visualized using Bio-Lumina, an ECL-type HRP chemiluminescent substrate (PB-L Productos Bio-Lógicos, Argentina; cat. RA0102; reported sensitivity ~10 pg), according to the manufacturer’s instructions. Band intensity was quantified using optical densitometry with ImageJ/Fiji software (National Institutes of Health, USA). After densitometry of immunoblots, values of the RD group were normalized to 100%, and both SRD and SRD + Ca were expressed relative to this. The protein levels were normalized to those of β-actin, detected using a mouse monoclonal anti-β-actin antibody (sc-47778, Santa Cruz Biotechnology; 1:50 dilution).

### 2.11 Transmission electron microscopy

Heart tissue samples were fixed in 4% (w/v) paraformaldehyde and 0.25% (v/v) glutaraldehyde. After 4 h, samples were post-fixed in 1% w/v osmium tetroxide for 30 min. After dehydration in an ethanol gradient, samples were contrasted with 5% w/v uranyl acetate and embedded in Durcupan (Fluka AG Chemische Fabrik, Buchs, SG, Switzerland).

Ultrathin sections were obtained with a Reichert-Jung Ultracut E ultramicrotome (Leica Microsystems GmbH, Wetzlar, Germany) and stained with lead citrate (61). Images were acquired on a Zeiss 109 Transmission Electron Microscope (TEM) (Carl Zeiss, Oberkochen, Germany) and photographed with a Gatan CCD camera (Pleasanton, CA, USA).

### 2.12 Statistical analysis

Results were expressed as mean ± SEM. Statistical comparisons were made transversely between different dietary groups. The data were evaluated for normality and homogeneity of variances using the Shapiro–Wilk and Levene tests, respectively, prior to the application of parametric tests. The statistical difference between groups (RD, SRD and SRD + Ca) was determined by one-way ANOVA followed by Tukey’s multiple comparisons test. Statistical analyses were performed using GraphPad Prism software, v8 (GraphPad Software, San Diego, CA, USA). Systolic and diastolic blood pressure, recorded weekly throughout the experimental period, were analyzed by two-way repeated-measures ANOVA, with diet (RD, SRD, SRD + Ca) as the between-subjects factor and time (weeks) as the within-subjects repeated factor. The three dietary groups were compared with each other within each time point using Tukey’s multiple comparisons test.

## 3 Results

### 3.1 Hemodynamic, electrocardiographic and metabolic and cardiac injury parameters

SRD-fed rats exhibited a progressive increase in both systolic (sBP) and diastolic blood pressure (dBP) compared with RD animals, becoming significant from week 2 onward and remaining elevated at week 3 (Figures 2A,B). Representative ECG traces did not reveal overt abnormalities suggestive of altered cardiac electrical conduction (Figure 2C). No statistically significant differences were observed between the groups in any of the quantified electrocardiographic parameters. Also, as shown in Table 2, no significant differences were observed in initial body weight, final body weight, daily food intake, heart weight, or relative heart weight among the experimental groups. Regarding serum metabolic parameters, SRD administration induced significant increases in serum uric acid, TG, NEFA, total cholesterol, and LDL-cholesterol levels, together with a significant reduction in HDL-cholesterol compared with the RD group, whereas serum glucose levels remained unchanged among groups. In addition, cardiac injury markers were significantly increased in the SRD group, as reflected by higher CK-MB and LDH levels compared with the RD group. Concomitant CO administration attenuated the development of the increase in both sBP and dBP, maintaining values close to those observed in RD rats throughout the experimental period. Quantitative ECG parameters in the SRD + Ca group remained comparable to those of the other groups. Likewise, concomitant CO administration prevented or attenuated the metabolic alterations induced by SRD. In particular, serum uric acid, TG, total cholesterol, and LDL-cholesterol levels were significantly reduced compared with SRD group, reaching values similar to those observed in RD animals, whereas NEFA levels were also significantly decreased but remained above those observed in the RD group. In addition, the SRD-induced decrease in HDL-cholesterol was prevented in the SRD + Ca group. Moreover, CO administration prevented the increase in cardiac injury markers, significantly decreasing CK-MB and LDH levels compared with SRD animals and with values close to those observed in the RD group.

**Figure 2:**
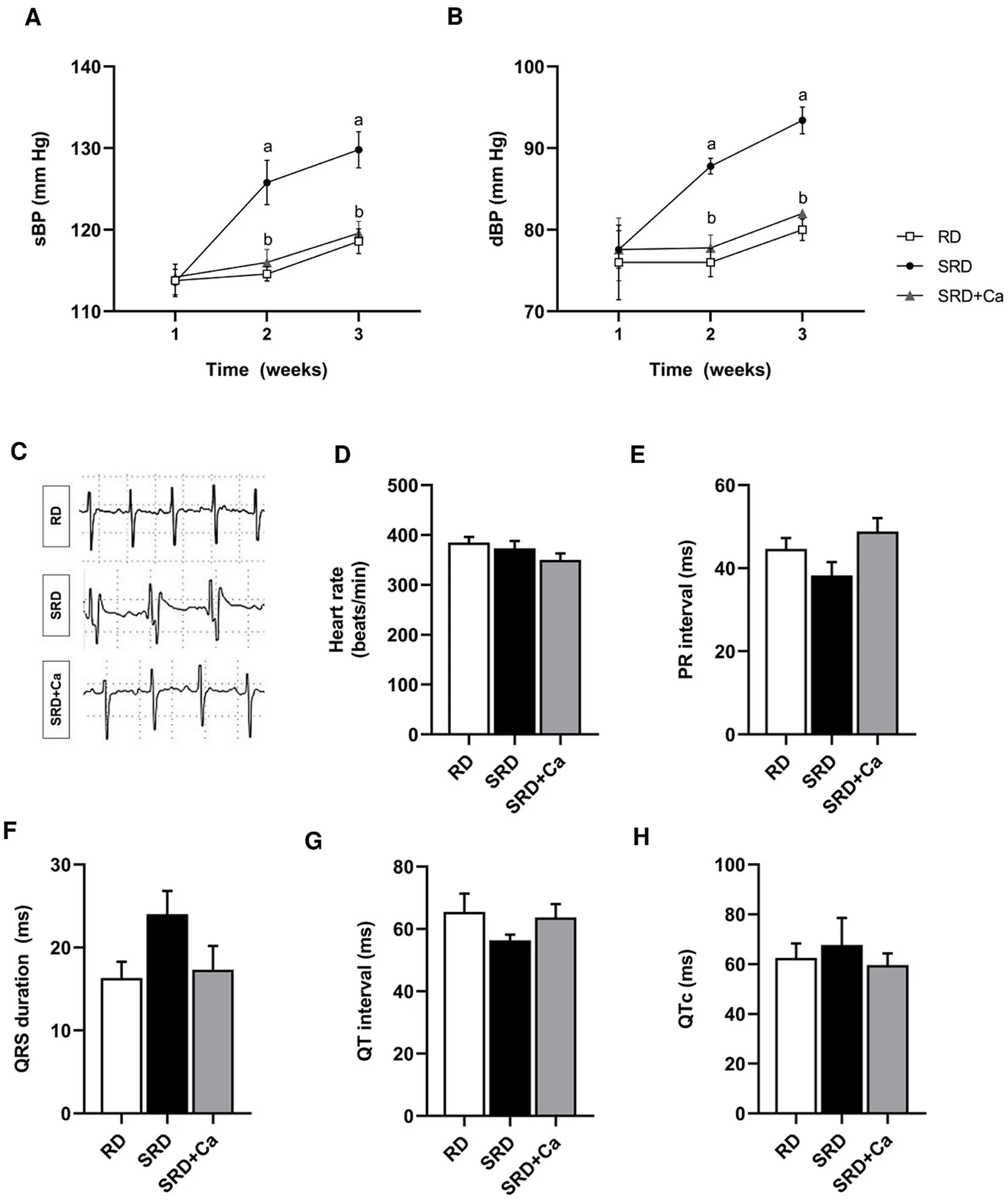
Hemodynamic alterations and representative electrocardiographic traces in rats fed a reference diet (RD), a sucrose-rich diet (SRD), or SRD plus CO (SRD + Ca). **(A)** Systolic blood pressure (SBP) and **(B)** diastolic blood pressure (DBP) recorded weekly throughout the experimental period. **(C)** Representative lead II electrocardiographic traces obtained at the end of the experimental period. **(D)** Heart rate. **(E)** PR interval. **(F)** QRS duration. **(G)** QT interval. **(H)** Corrected QT interval (QTc). Data are expressed as mean ± SEM (*n* = 6). Statistical analysis was performed by one-way ANOVA followed by Tukey’s multiple comparisons test. Quantitative data are expressed as mean ± SEM (*n* = 6). Statistical differences were evaluated by one-way ANOVA followed by Tukey’s *post hoc* test. Values that do not share the same superscript letter are significantly different (*p* < 0.05); the absence of letters indicates no significant differences among the experimental groups.

**Table 2:** Body weight, food intake, serum metabolites, heart weight, relative weight and cardiac injury markers.

Parameter	RD	SRD	SRD+Ca
Initial body weight (g)	178.0 ± 2.5	180.3 ± 4.3	179.7 ± 2.3
Final body weight (g)	269.92 ± 4.85	267.3 ± 5.9	272.2 ± 4.1
Food intake (g/day)	16.82 ± 0.35	16.87 ± 0.27	16.90 ± 0.43
Serum metabolites			
Glucose (mM)	8.51 ± 0.15	8.44 ± 0.16	8.42 ± 0.19
Uric acid (μM)	63.97 ± 6.12^b^	120.30 ± 6.07^a^	77.55 ± 3.75^b^
TG (mM)	1.32 ± 0.09^b^	3.08 ± 0.16^a^	1.23 ± 0.07^b^
NEFA (μM)	401.9 ± 38.9^c^	827.9 ± 28.7^a^	615.1 ± 55.3^b^
Total Cholesterol (mM)	2.02 ± 0.10^b^	2.64 ± 0.09^a^	2.12 ± 0.18^b^
HDL-Cholesterol (mM)	1.11 ± 0.04^a^	0.70 ± 0,12^b^	1.03 ± 0.09^a^
LDL-Cholesterol (mM)	0.09 ± 0.01^b^	0.12 ± 0.01^a^	0.09 ± 0.01^b^
Cardiac parameters			
Heart weight (g)	0.95 ± 0.01	0.97 ± 0.02	0.93 ± 0.01
Relative heart weight (g/100 g)	0.34 ± 0.01	0.34 ± 0.01	0.33 ± 0.01
CK-MB (U/L)	385.91 ± 40.64^b^	580.63 ± 45.13^a^	355.83 ± 16.69^b^
LDH (U/L)	0.54 ± 0.03^b^	0.66 ± 0.03^a^	0.53 ± 0.03^b^

Values are expressed as mean ± SEM (*n* = 6). Within each row, values that do not share the same letter are significantly different (*p* < 0.05), as determined by one-way ANOVA followed by Tukey’s multiple comparisons test. RD: reference diet; SRD: sucrose-rich diet; SRD+Ca: SRD plus CO.

### 3.2 Cardiac lipid accumulation and histological features

SRD-fed rats showed a significant increase in cardiac triglyceride content compared with RD animals (Figure 3B). In line with this, histological analysis revealed marked alterations in myocardial architecture in the SRD group, including disorganization of muscle fibers, cardiomyocyte nuclear deformation, perinuclear vacuolization, and focal interstitial hemorrhage, whereas these features were not observed in RD animals (Figure 3A). Daily CO administration prevented cardiac TG accumulation, maintaining values comparable to those of the RD group. Consistently, myocardial architecture was preserved in SRD + Ca animals, with no evidence of the structural alterations observed in SRD-fed rats.

**Figure 3:**
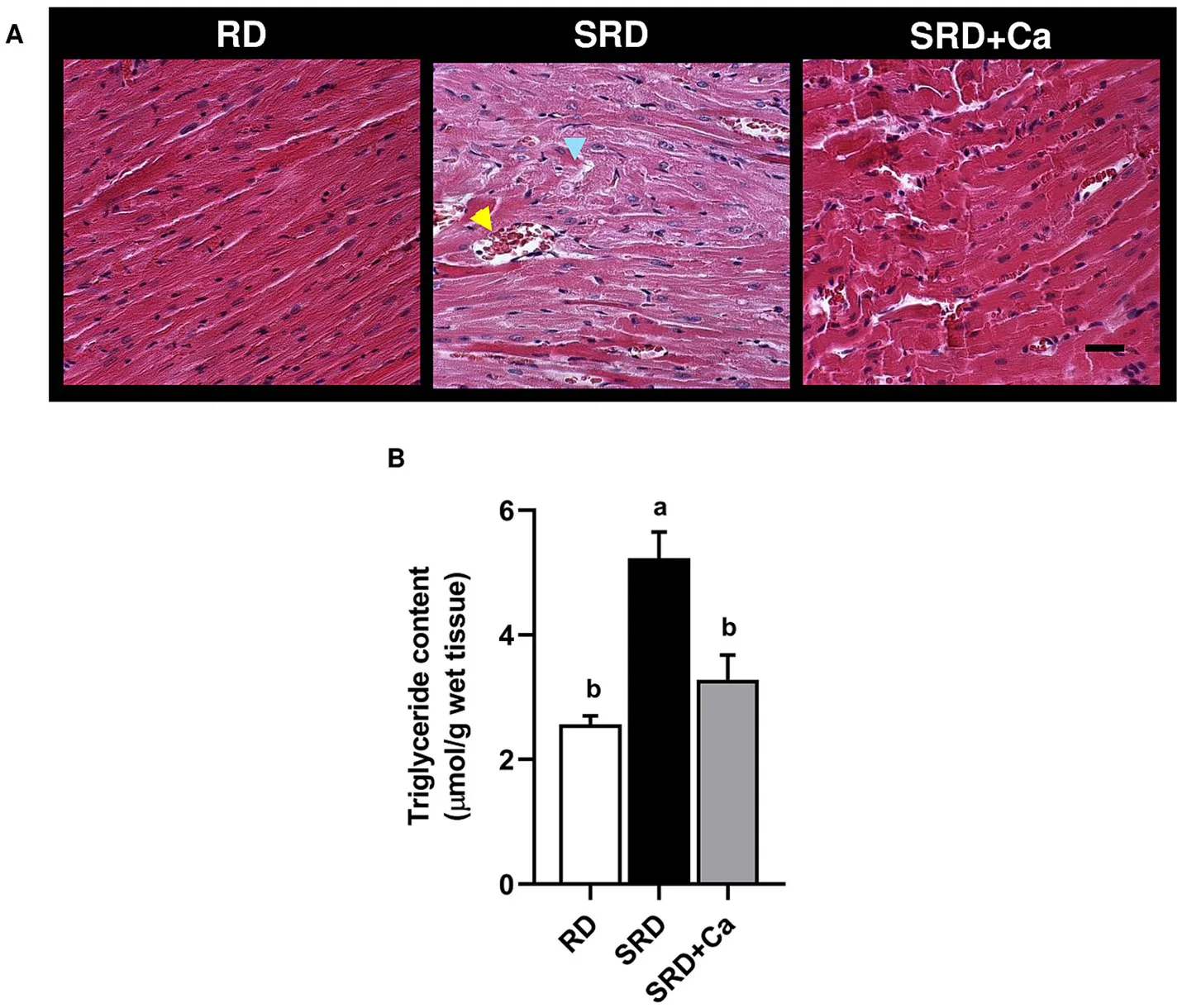
Cardiac histological features and TG content in rats fed a reference diet (RD), a sucrose-rich diet (SRD), or SRD plus CO (SRD + Ca). **(A)** Representative photomicrographs of myocardial sections (x200 magnification). The SRD group exhibited signs of myocardial structural alteration, including myofiber disorganization, cardiomyocyte nuclear deformation, perinuclear vacuolization (blue arrow), and vascular congestion (yellow arrow). These histological alterations were less evident in the SRD + Ca group, which showed a more preserved myocardial architecture. Scale bar = 50 μm. **(B)** Cardiac TG content. Quantitative data are expressed as mean ± SEM (*n* = 6). Statistical differences were evaluated by one-way ANOVA followed by Tukey’s *post hoc* test. Values that do not share the same superscript letter are significantly different (*p* < 0.05); the absence of letters indicates no significant differences among the experimental groups.

### 3.3 Cardiac lipid metabolism-related parameters

SRD feeding significantly increased the relative mRNA expression of both SREBP-1c and PPARα compared with RD animals (Figures 4A,B). In parallel, SRD-fed rats exhibited significantly higher activities of key enzymes involved in lipid metabolism, including ACC, FAS, malic enzyme, G6PDH, and CPT-1 (Figures 4C–G). Cannabis oil administration prevented the SRD-induced increase in SREBP-1c expression, with values remaining comparable to those observed in RD animals. In contrast, PPARα expression remained elevated in this group. Daily CO administration also prevented the SRD-induced increases in ACC, FAS, malic enzyme, G6PDH, and CPT-1 activities, maintaining values not significantly different from those observed in RD animals.

**Figure 4:**
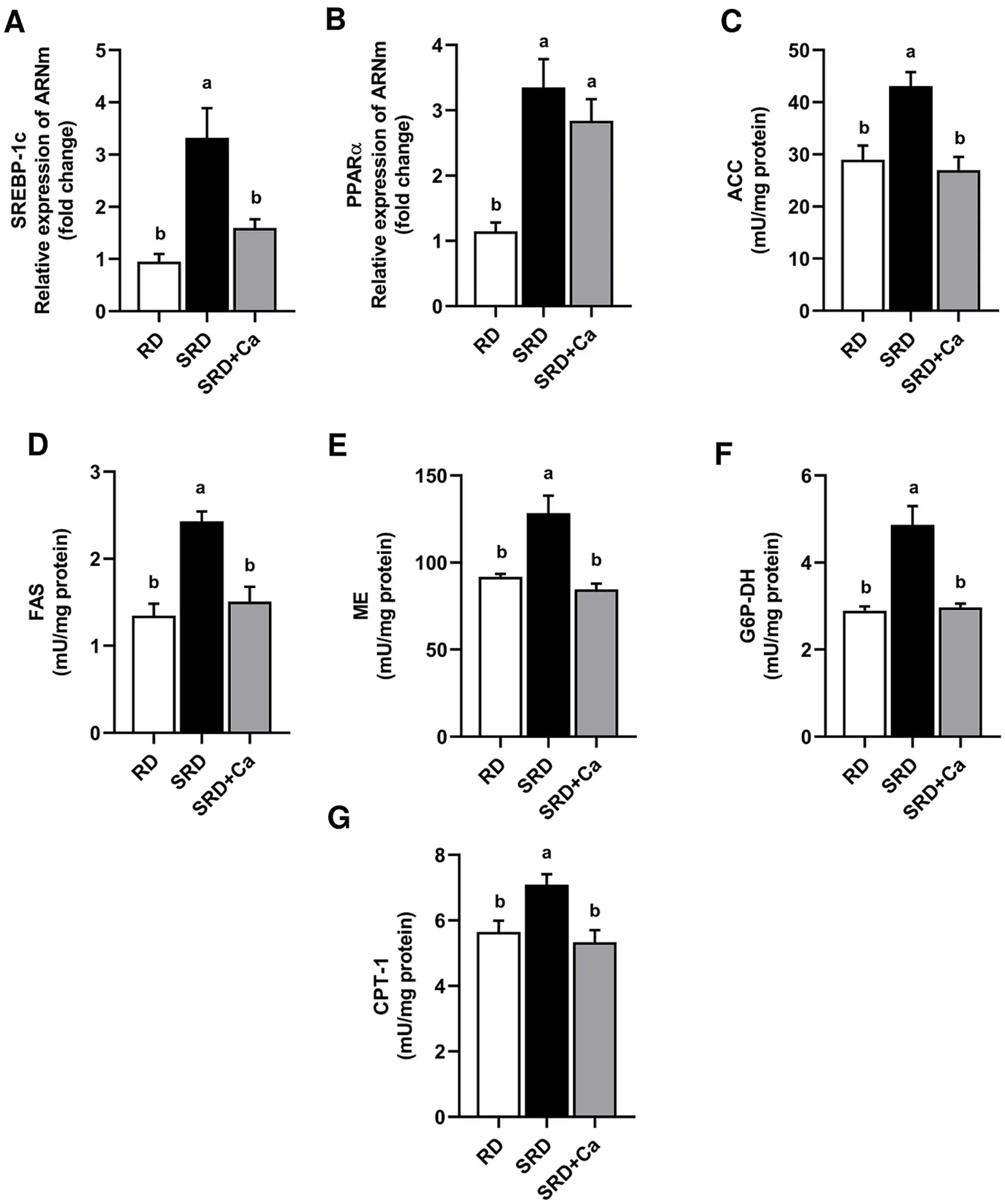
Cardiac lipid metabolism-related parameters in rats fed a reference diet (RD), sucrose-rich diet (SRD) and SRD plus CO (SRD + Ca). **(A)** Relative SREBP-1c mRNA expression. **(B)** Relative PPARα mRNA expression. **(C)** Acetyl-CoA carboxylase (ACC) activity. **(D)** Fatty acid synthase (FAS) activity. **(E)** Malic enzyme (ME) activity. **(F)** Glucose-6-phosphate dehydrogenase (G6P-DH) activity. **(G)** Carnitine palmitoyltransferase-1 (CPT-1) activity. Gene expression data are expressed as fold change relative to the RD group. Quantitative data are expressed as mean ± SEM (*n* = 6). Statistical differences were evaluated by one-way ANOVA followed by Tukey’s *post hoc* test. Values that do not share the same superscript letter are significantly different (*p* < 0.05); the absence of letters indicates no significant differences among the experimental groups.

### 3.4 Cardiac oxidative stress and antioxidant related parameters

As shown in Table 3, SRD-fed rats exhibited a marked disruption of cardiac redox homeostasis compared with RD animals, as evidenced by significantly increased ROS levels, together with reduced CAT, GR, and GPx activities, lower GSH content, and decreased Nrf2 mRNA expression. Daily CO prevented these alterations, maintaining all evaluated parameters at values comparable to those observed in the RD group.

**Table 3:** Cardiac oxidative stress markers and antioxidant defense-related parameters.

Parameter	RD	SRD	SRD+Ca
CAT activity (U/mg protein)	233.97 ± 15.97^a^	156.29 ± 7.62^c^	190.98 ± 6.68^b^
GR activity (U/mg protein)	9.88 ± 0.90^a^	4.01 ± 0.62^b^	8.65 ± 0.79^a^
GPx activity (nmol/mg protein)	110.37 ± 5.56^a^	62.68 ± 3.88^b^	106.62 ± 9.09^a^
GSH content (nmol/mg protein)	60.67 ± 3.96^a^	48.57 ± 2.17^b^	57.84 ± 1.78^a^
ROS (UF relative to RD)	1.04 ± 0.08^b^	1.61 ± 0.12^a^	1.12 ± 0.03^b^
Nrf2 (relative mRNA expression)	0.97 ± 0.06^a^	0.41 ± 0.05^b^	1.10 ± 0.13^a^

Values are expressed as mean ± SEM, *n* = 6. Values in a line that do not share the same superscript letter are significantly different (*p* < 0.05) when one variable at a time was compared by one-way ANOVA followed by Tukey’s multiple comparisons test. Reference diet (RD), sucrose-rich diet (SRD) or SRD plus CO (SRD+Ca).

Consistent with this redox imbalance, SRD feeding also enhanced cardiac lipid peroxidation, as evidenced by increased TBARS levels and greater accumulation of 4-HNE-modified proteins in cardiac tissue (Figures 5A–C). Cannabis oil co-administration prevented this lipoperoxidative response, maintaining TBARS levels comparable to those observed in RD animals and reducing the immunoreactivity of 4-HNE-modified proteins to values comparable to those observed in RD animals.

**Figure 5:**
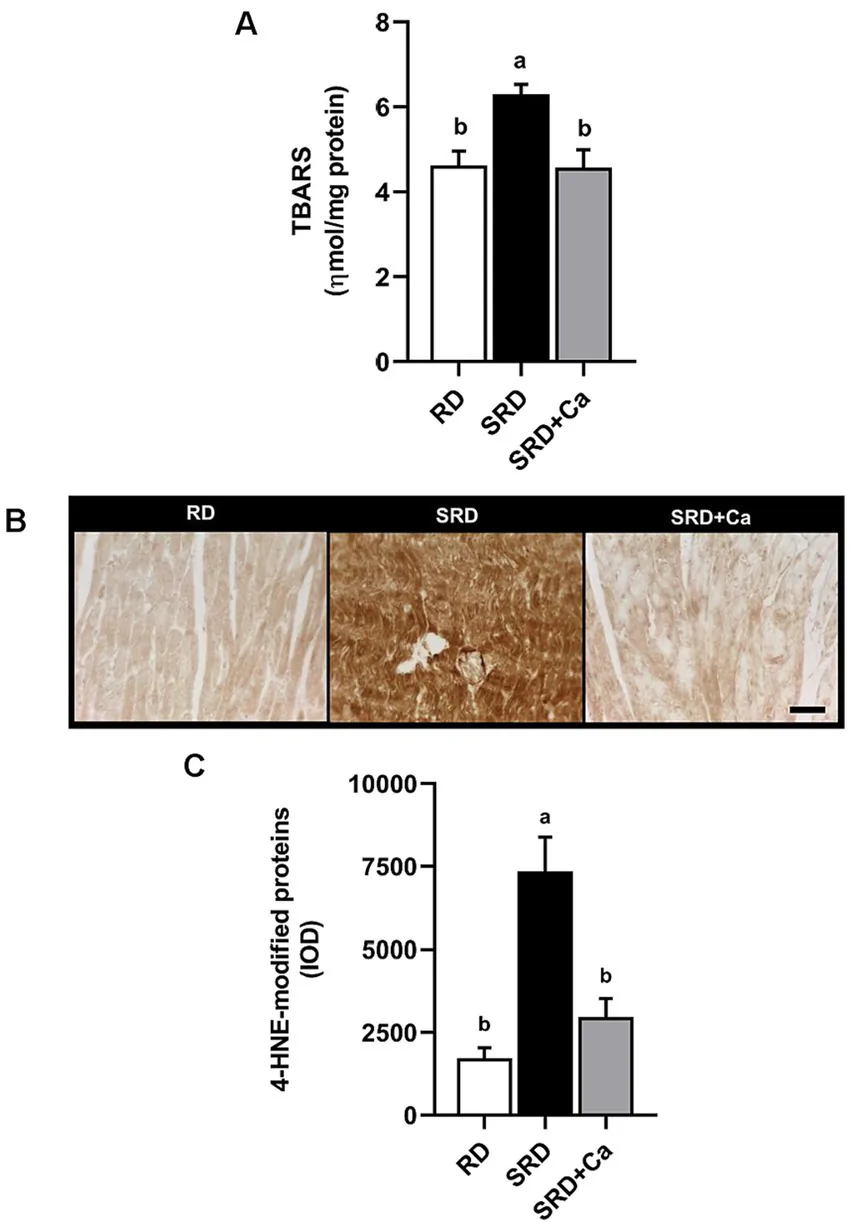
Cardiac lipid peroxidation markers in rats fed a reference diet (RD), sucrose-rich diet (SRD) and SRD plus CO (SRD + Ca). **(A)** Cardiac thiobarbituric acid reactive substances (TBARS) levels. **(B)** Representative photomicrographs of 4-HNE-modified protein immunoreactivity in cardiac sections acquired using a 40 × objective. Immunoreactivity was visualized using 3,3′-diaminobenzidine (DAB) as the chromogenic substrate, without counterstaining. Scale bar = 50 μm **(C)** Quantitative immunohistochemical analysis of immunoreactivity of 4-HNE-modified proteins are expressed as Integrated optical density (IOD). Quantitative data are expressed as mean ± SEM (*n* = 6). Statistical differences were evaluated by one-way ANOVA followed by Tukey’s *post hoc* test. Values that do not share the same superscript letter are significantly different (*p* < 0.05); the absence of letters indicates no significant differences among the experimental groups.

### 3.5 Cardiac inflammatory and endothelial activation-related parameters

SRD feeding significantly increased the relative mRNA expression of F4/80, TNFα, MCP-1, iNOS, and NF-κB in the heart compared with RD animals, indicating the establishment of a pro-inflammatory transcriptional profile (Figure 6A). In parallel, SRD markedly reduced cardiac IL-10 mRNA expression, suggesting an impairment of anti-inflammatory counter-regulatory signaling (Figure 6B). Cannabis oil administration attenuated the SRD-induced pro-inflammatory response, reducing the mRNA expression of F4/80, TNFα, MCP-1, iNOS, and NF-κB relative to the SRD group. Notably, although F4/80 and iNOS mRNA expression were decreased by CO, both remained significantly higher than in RD animals. In contrast, TNFα, MCP-1, and NF-κB mRNA expression did not differ significantly from values observed in RD animals. However, CO did not prevent the SRD-induced decrease in IL-10 mRNA expression, which remained significantly lower than in RD animals and did not differ from the SRD group. In contrast to the other inflammatory and endothelial activation-related genes, cardiac PAI-1 mRNA expression was not significantly modified by the sucrose-rich diet and did not differ among the three experimental groups (RD, SRD and SRD + Ca) (Figure 6A).

**Figure 6:**
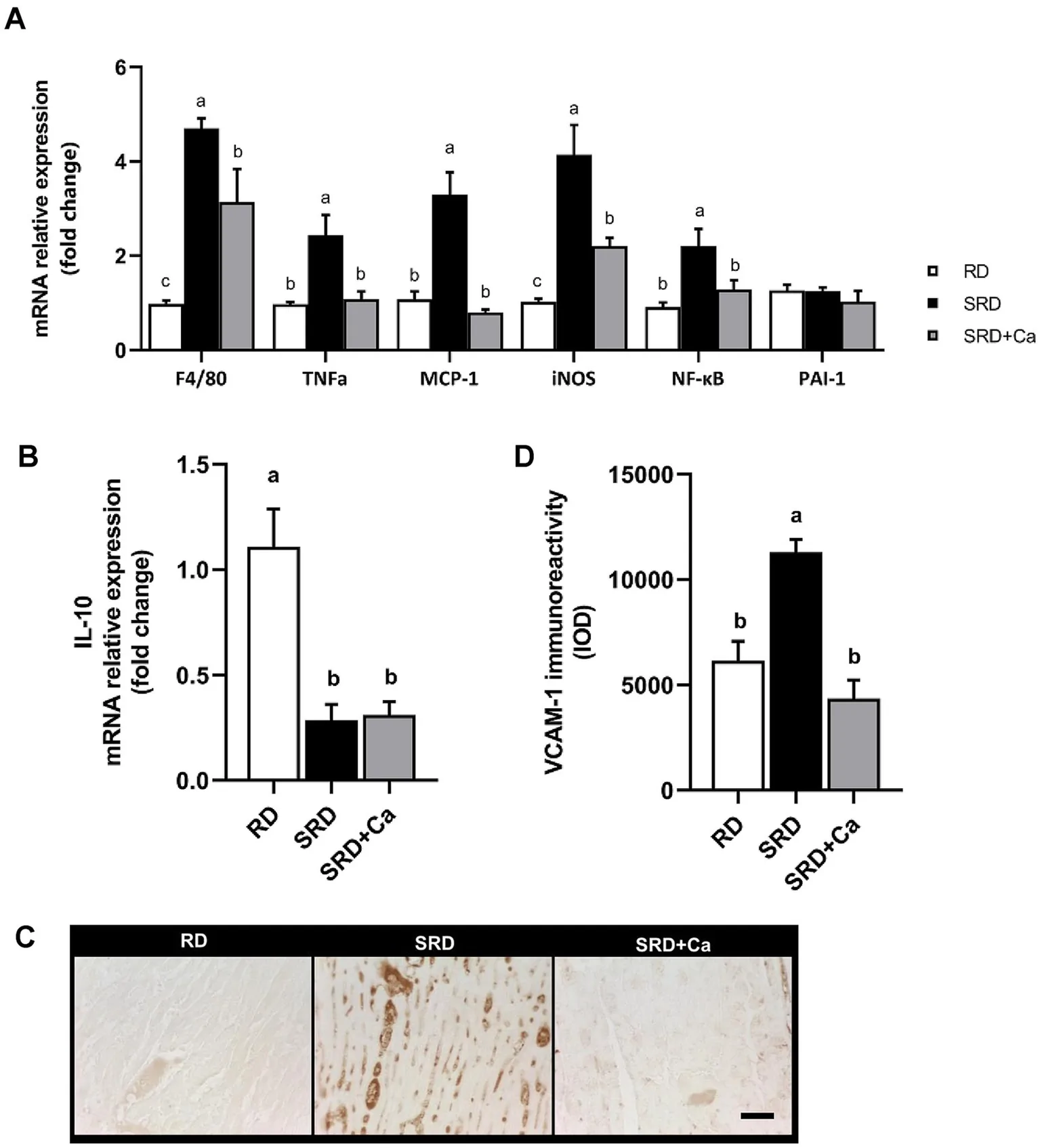
Cardiac inflammatory and endothelial activation-related parameters in rats fed a reference diet (RD), a sucrose-rich diet (SRD), or SRD plus CO (SRD + Ca). **(A)** Relative mRNA expression of the inflammatory markers F4/80, TNF-α, MCP-1, iNOS, NF-κB and PAI-1. **(B)** Relative mRNA expression of IL-10. Gene expression data are expressed as fold change relative to the RD group. **(C)** Representative photomicrographs of VCAM-1 immunoreactivity in cardiac sections acquired using a 40 × objective. Immunoreactivity was visualized using 3,3′-diaminobenzidine (DAB) as the chromogenic substrate, without counterstaining. Scale bar = 50 μm. **(D)** Quantitative immunohistochemical analysis of VCAM-1 immunoreactivity. Values (mean ± SEM, *n* = 6) are expressed as Integrated optical density (IOD). Quantitative data are expressed as mean ± SEM (*n* = 6). Statistical differences were evaluated by one-way ANOVA followed by Tukey’s *post hoc* test. Values that do not share the same superscript letter are significantly different (*p* < 0.05); the absence of letters indicates no significant differences among the experimental groups.

Endothelial activation was further assessed by VCAM-1 immunoreactivity in cardiac tissue. SRD-fed rats exhibited a significant increase in VCAM-1 immunostaining intensity compared with RD animals, whereas CO co-administration prevented this increase, maintaining VCAM-1 IOD values within the range observed in the RD group (Figure 6C).

### 3.6 Cardiac fibrotic parameters

Figure 6 shows that SRD feeding significantly increased cardiac collagen deposition, as evidenced by picrosirius red staining under bright-field and polarized light microscopy and confirmed by quantitative analysis (Figures 7A–C), together with increased TGF-β mRNA expression (Figure 7D). Cannabis oil attenuated both collagen accumulation and TGF-β upregulation, maintaining values close to those observed in RD animals.

**Figure 7:**
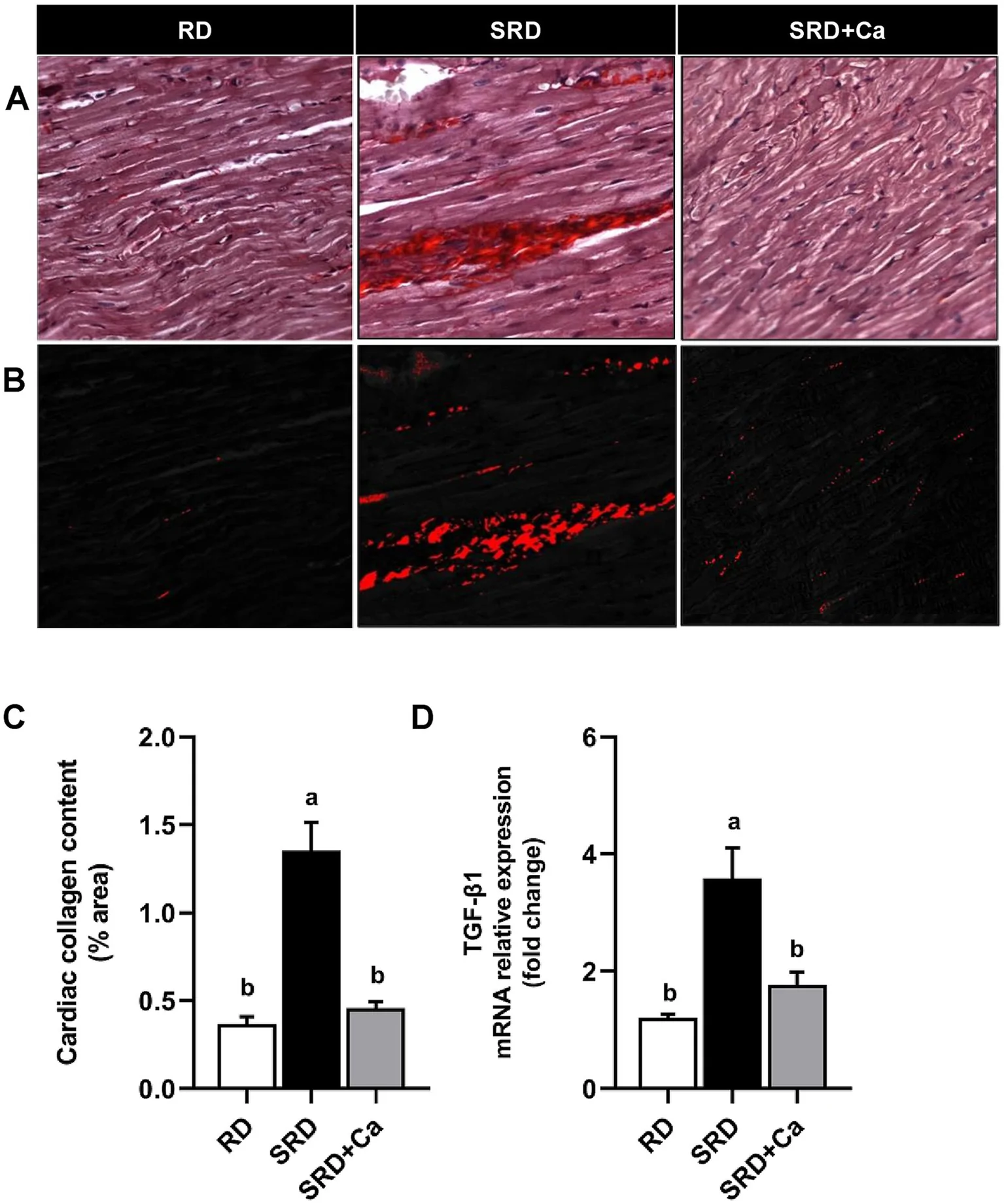
Cardiac fibrotic remodeling related parameters in rats fed a reference diet (RD), sucrose-rich diet (SRD) and SRD plus CO (SRD + Ca). **(A)** Picrosirius-stained histological sections of the heart. Abnormal collagen deposition is observed around cardiomyocytes and blood vessels in the SRD group. **(B)** Cardiac collagen-positive area (% of total tissue area). Scale bar: 50 μm. **(C)** Quantification of collagen-positive areas was calculated relative to the total areas in heart sections. **(D)** Relative mRNA expression of TGF-β. Data are expressed as mean ± SEM (*n* = 6). Statistical differences were evaluated by one-way ANOVA followed by Tukey’s *post hoc* test. Values that do not share the same superscript letter are significantly different (*p* < 0.05); the absence of letters indicates no significant differences among the experimental groups.

### 3.7 Cardiac ultrastructural features

Transmission electron microscopy (TEM) revealed a well-preserved myocardial ultrastructure in RD rats, characterized by the normal organization of sarcomeres, intact mitochondria with preserved cristae, and regular nuclear morphology (Figure 8). In contrast, SRD-fed animals displayed marked ultrastructural abnormalities indicative of cellular damage and tissue remodeling. These alterations included prominent intracellular glycogen accumulation, disruption and disorganization of myofibrils, loss of sarcomeric alignment, and nuclei exhibiting condensed chromatin. In addition, increased collagen deposition was observed within the interstitial space, consistent with the development of myocardial fibrosis. Perivascular edema surrounding small-caliber vessels was also evident, indicating microvascular impairment and altered tissue homeostasis. Mitochondrial swelling and heterogeneous electron density were occasionally detected in cardiomyocytes from SRD-fed animals. Notably, the SRD + Ca group exhibited a substantial attenuation of these ultrastructural abnormalities, showed reduced perivascular edema, lower collagen accumulation, and greater preservation of myofibrillar architecture and cellular organization compared to SRD group.

**Figure 8:**
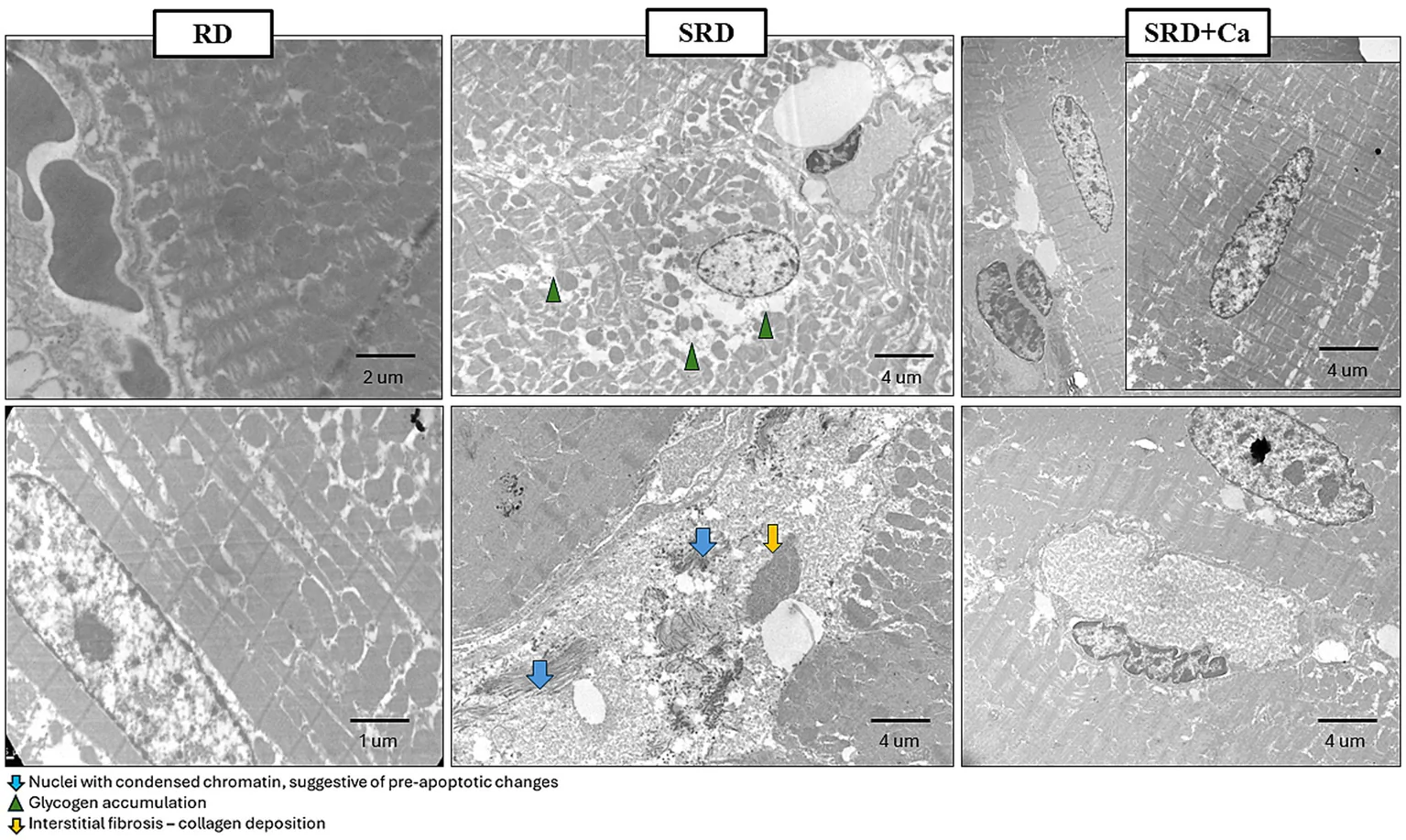
Representative transmission electron micrographs of cardiac tissue from rats fed a reference diet (RD), sucrose-rich diet (SRD) and SRD plus CO (SRD + Ca). SRD animals show marked ultrastructural alterations, including glycogen accumulation (green arrows), nuclei with condensed chromatin (blue arrows), and collagen-rich fibrotic areas (orange arrows). In the SRD + Ca group, myocardial ultrastructure appears better preserved, with reduced perivascular edema and improved cytoarchitectural organization compared with the SRD group.

### 3.8 Cardiac cannabinoid receptor protein expression

SRD feeding significantly increased cardiac CB_1_R and CB_2_R protein expression relative to RD animals (Figure 9). Daily CO administration prevented both changes, maintaining receptor protein levels close to those observed in the RD group.

**Figure 9:**
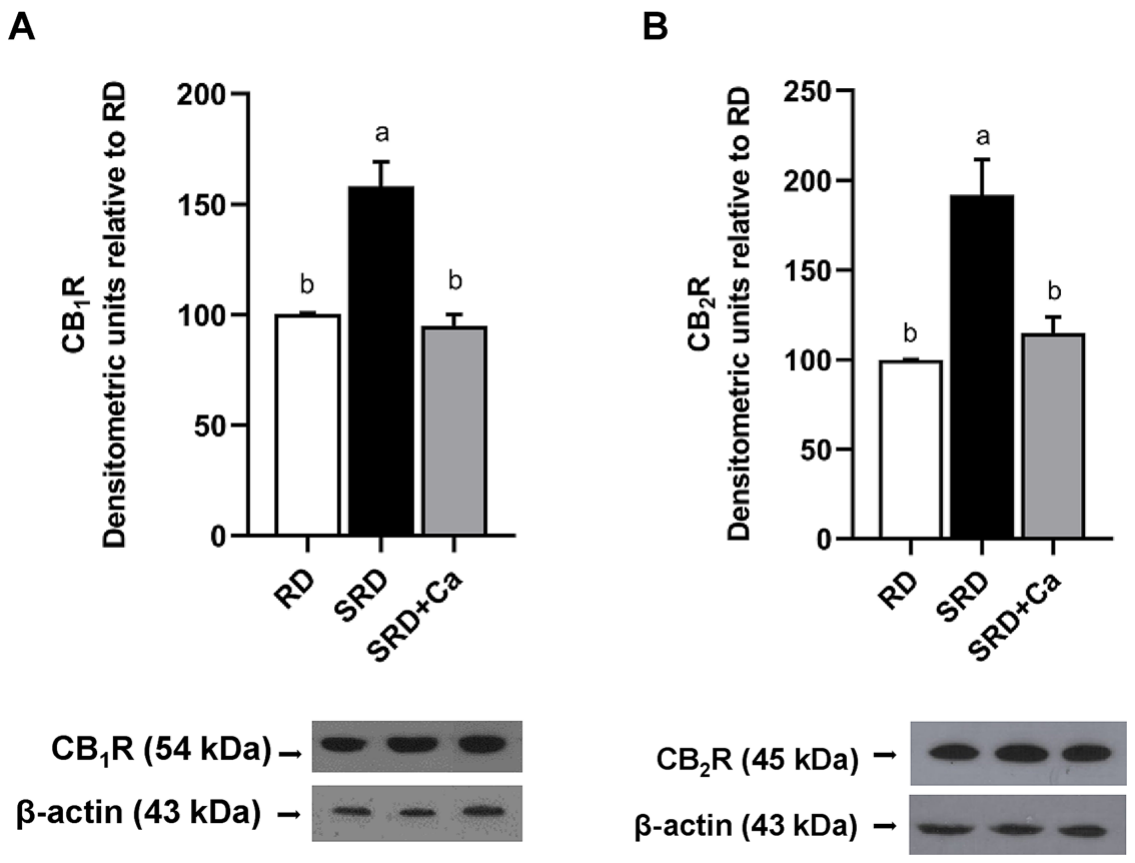
Cardiac cannabinoid receptor protein expression in rats fed a reference diet (RD), sucrose-rich diet (SRD) and SRD plus CO (SRD + Ca). **(A)** Cardiac CB1 receptor (CB_1_R) protein expression determined by western blot and corresponding densitometric analysis relative to the RD group. **(B)** Cardiac CB2 receptor (CB_2_R) protein expression determined by western blot and corresponding densitometric analysis relative to the RD group. Representative immunoblots for CB_1_R, CB_2_R, and β-actin are shown below each graph. Data are expressed as mean ± SEM (*n* = 6). Statistical differences were evaluated by one-way ANOVA followed by Tukey’s *post hoc* test. Values that do not share the same superscript letter are significantly different (*p* < 0.05); the absence of letters indicates no significant differences among the experimental groups.

## 4 Discussion

The present study shows that short-term SRD feeding induced a coordinated pattern of early myocardial alterations, including biochemical changes compatible with cardiac injury, lipid accumulation, oxidative stress, inflammatory and endothelial activation, ultrastructural disorganization, and increased cardiac protein expression of cannabinoid receptors CB_1_R and CB_2_R. Notably, these alterations developed in the absence of overt electrocardiographic abnormalities, suggesting that metabolic and structural injury may precede detectable electrical dysfunction in this model.

The rise in systolic and diastolic blood pressure, together with elevated CK-MB and LDH activities, was compatible with early myocardial involvement after short-term SRD feeding. In agreement with our findings, increased levels of these enzymes have been reported in experimental models of metabolic dysfunction, including diet-induced and diabetic conditions, supporting their use as biochemical indicators compatible with cardiac damage when interpreted together with tissue-level alterations (62, 63). In contrast to the biochemical and structural alterations observed, global cardiac electrical activity remained statistically unchanged after three weeks of SRD, as none of the electrocardiographic parameters assessed showed significant differences between groups. Previous studies in short-duration metabolic models have suggested that biochemical and structural alterations may become detectable before electrophysiological changes (64, 65). In this context, the absence of significant electrocardiographic differences in the present study is compatible with the possibility that the metabolic, biochemical, and structural alterations detected at this early time point precede detectable changes in global cardiac electrical activity in this model. Importantly, the preservation of ECG parameters in the SRD + Ca group further suggests that this intervention does not exert detectable cardiotoxic effects under the present experimental conditions.

A central finding of this study was the early establishment of early cardiac alterations associated with myocardial lipid overload in SRD-fed rats. This was evidenced by increased cardiac triglyceride content, indicating early lipid deposition within the heart. Previous studies from our group demonstrated cardiac lipid accumulation in rats subjected to chronic SRD feeding, in association with dyslipidemia and hypertension (27, 29). Our data extend those observations by showing that myocardial lipid deposition can already be detected at much earlier stages of sucrose overload. This lipid accumulation was accompanied by histological alterations, including myofiber disorganization, nuclear deformation, perinuclear vacuolization, and occasional focal interstitial hemorrhagic areas, suggesting that metabolic overload is accompanied by early structural myocardial alterations. Similar features have been described in experimental settings of cardiac lipotoxicity, where excessive lipid deposition is associated with cytoarchitectural disruption and early tissue damage (66, 67).

At the metabolic level, this profile was supported by increased activities of key lipogenic enzymes and SREBP-1c upregulation, consistent with activation of a myocardial lipogenic program. In parallel, CPT-1 activity and PPARα expression were also increased, which at first sight would suggest an adaptive upregulation of the fatty acid oxidation machinery in response to elevated lipid availability. However, because oxidative flux was not directly assessed, these changes should be interpreted as a transcriptional and enzymatic response rather than as evidence of increased β-oxidation per se. Critically, this upregulation failed to restore lipid homeostasis, as TG continued to accumulate within the myocardium. In the myocardium, PPARα acts as a master regulator of fatty acid oxidation and substrate handling, and its activity is canonically associated with cardioprotection and with the preservation of cardiomyocyte cytoarchitecture under stress (68, 69). The fact that, in our model, PPARα induction coexisted with lipid overload and structural injury rather than preventing them suggests that this response does not fulfill its canonical protective role, but instead represents a maladaptive adaptation to a sustained sucrose-driven lipid surplus. This myocardial pattern parallels the lipid-handling alterations previously reported by our group in the chronic dietary model (27), indicating that the cardiac PPARα response is engaged from early stages and sustained into chronicity. At this same early timepoint and under the same dietary challenge, the liver displayed the opposite pattern, with downregulation of CPT-1 and PPARα (49). This divergence underscores a tissue- and pathway-specific handling of the sucrose-derived lipid surplus, in which heart and liver mount qualitatively opposite transcriptional responses to the same systemic overload. If sustained, this myocardial configuration may favor excessive reliance on fatty acid metabolism, oxidative stress, and reduced metabolic flexibility, thereby contributing to early myocardial remodeling.

The alterations in cardiac lipid metabolism and TG accumulation observed in SRD-fed rats were accompanied by oxidative stress. Under conditions of lipid oversupply, excessive mitochondrial fatty acid flux is known to favor ROS generation and lipid peroxidation, as reflected here by increased ROS and TBARS levels and the accumulation of 4-HNE-modified proteins. In agreement with this, previous studies have reported increased lipid peroxidation and impaired antioxidant capacity in cardiac tissue from sucrose-fed rats with MetS (70, 71). Our findings further support an impaired antioxidant defense profile, as evidenced by GSH depletion, reduced antioxidant enzyme activities, and downregulation of Nrf2. This is mechanistically relevant because Nrf2 governs the transcription of antioxidant and detoxifying genes, so that its downregulation is expected to compromise the glutathione system. Indeed, GSH and antioxidant enzymes such as CAT, GPx, and GR constitute a major component of the cardiac antioxidant network, and their depletion favors the accumulation of oxidant species and propagation of tissue injury, a concept highlighted by More et al. (34) in the context of myocardial oxidative damage. Beyond direct injury to proteins and DNA, excessive ROS may also activate matrix remodeling pathways and pro-hypertrophic and pro-apoptotic signaling, thereby contributing to fibrosis and structural deterioration (72).

Consistent with this oxidative context, the inflammatory and remodeling-related data indicate that SRD feeding rapidly establishes a pro-inflammatory myocardial environment. Higher myocardial mRNA expression of NF-κB, TNFα, MCP-1, and the macrophage marker F4/80 was consistent with activation of inflammatory signaling and a macrophage-related inflammatory response, whereas increased TGF-β mRNA expression, together with concomitant myocardial collagen deposition, supports early engagement of profibrotic signaling and extracellular matrix remodeling. Given the short duration of the dietary intervention, these findings are more appropriately interpreted as early collagen accumulation and matrix remodeling rather than as evidence of fully established myocardial fibrosis. This pro-inflammatory shift was accompanied by a reduction in the anti-inflammatory cytokine IL-10, further reflecting an imbalance toward a pro-inflammatory state. In contrast, cardiac PAI-1 mRNA expression was not significantly modified by the sucrose-rich diet. Although PAI-1 is a TGF-β–responsive, profibrotic and prothrombotic mediator frequently up-regulated in advanced cardiac remodeling, its lack of induction at this early timepoint suggests that not all remodeling-associated mediators are engaged simultaneously, and that PAI-1 up-regulation may require a more sustained or advanced stage of injury than that reached after three weeks of SRD feeding. This interpretation should be taken with caution, since a non-significant change does not exclude subtle alterations below the detection threshold of the present design. NF-κB is particularly relevant in this context because it transcriptionally regulates multiple cytokines, inducible enzymes, and adhesion molecules, thereby linking oxidative stress to immune activation, endothelial activation, and remodeling (73, 74). In this line, increased VCAM-1 immunoreactivity, together with higher iNOS expression, supports the early establishment of endothelial activation associated with the inflammatory myocardial pattern. Taken together, these results indicate that short-term SRD feeding is sufficient to trigger an integrated redox–inflammatory–endothelial–profibrotic response rather than isolated metabolic alterations.

This integrated response is well recognized in experimental cardiometabolic disorders, where oxidative stress and NF-κB-dependent signaling contribute to macrophage-related inflammatory responses, endothelial activation, and extracellular matrix remodeling (64, 65). In our model, the molecular evidence of redox imbalance and inflammatory/profibrotic signaling was paralleled by ultrastructural abnormalities. TEM analysis revealed intracellular glycogen accumulation, myofibrillar disorganization with loss of sarcomeric alignment, nuclei with condensed chromatin, interstitial collagen deposition, and perivascular edema around small-caliber vessels. Mitochondrial swelling and heterogeneous electron density were also detected in SRD cardiomyocytes, providing a morphological counterpart to the lipotoxic and oxidative alterations described above, since the mitochondrion is both a primary site of fatty acid oxidation and a major source of ROS under lipid oversupply. These findings provide a morphological correlate for the biochemical and molecular alterations observed in SRD-fed rats and support the notion that short-term metabolic overload is sufficient to disrupt myocardial cytoarchitecture. Consistent with the rest of alterations, these ultrastructural abnormalities were markedly attenuated in the SRD + Ca group, which showed reduced perivascular edema, lower collagen accumulation, and better-preserved myofibrillar architecture.

Within this integrated cardiometabolic context, the increase in both CB_1_R and CB_2_R protein expression in SRD hearts suggests that sucrose-driven metabolic stress is associated with early remodeling of myocardial cannabinoid receptor expression. Components of the ECS are broadly distributed throughout the cardiovascular system, including cardiomyocytes, endothelial cells, and immune-related compartments, where they participate in the regulation of vascular tone, inflammatory responses, oxidative stress, and tissue remodeling (75). In general, CB_1_R signaling has been associated with ROS-dependent pro-inflammatory and profibrotic responses, whereas CB_2_R signaling is more frequently linked to immunomodulatory, antioxidant, and counter-regulatory mechanisms, although these effects are highly dependent on cell type, disease stage, and ligand context (33, 34, 42, 75, 76). The simultaneous increase in CB_1_R and CB_2_R in the hearts of SRD animals occurred in parallel with lipid accumulation and the redox, inflammatory and structural alterations observed. Since the present study evaluated protein abundance and non-activation of the receptor, downstream signaling or specific cell localization, these associations do not allow attributing a causal role to any of the receptors. The coexistence of the increase in CB_1_R with the upregulation of SREBP-1c is compatible with previous literature that relates CB_1_R signaling with lipogenic regulation, but a functional interaction between both processes was not evaluated in this study. Similarly, the increase in CB_2_R could represent an adaptive or compensatory response associated with the inflammatory context. Its concomitant increase with F4/80 also raises the possibility that part of the CB_2_R signal derives from cellular compartments of immune origin.

Importantly, concomitant daily oral administration of full-spectrum CO from the onset of SRD exposure prevented or attenuated multiple components of the developing myocardial response to SRD, including lipid accumulation, redox imbalance, accumulation of 4-HNE-modified proteins, inflammatory and endothelial activation, fibrotic signaling, structural alterations, and increased protein expression of CB_1_R and CB_2_R. However, this preventive effect was graded rather than uniform. The SRD-induced increases in TNF-α and MCP-1 were fully prevented, with values not significantly different from those observed in RD animals, whereas F4/80 and iNOS were significantly reduced relative to SRD but remained above RD levels, indicating partial attenuation. By contrast, the SRD-induced decrease in the anti-inflammatory cytokine IL-10 was not counteracted by CO, as IL-10 expression in the SRD + Ca group did not differ from that observed in SRD animals. At the tissue level, CO also prevented the SRD-induced increase in VCAM-1 immunoreactivity and in the accumulation of 4-HNE-modified proteins, supporting its ability to attenuate endothelial activation and lipid peroxidation-associated protein modification. Likewise, among lipid metabolism-related regulators, CO prevented the SRD-induced increase in SREBP-1c expression but only partially prevented the increase in PPARα. The attenuation of TGF-β upregulation, together with the reduction in myocardial collagen deposition in SRD + Ca animals, further suggests that CO attenuated the early extracellular matrix remodeling associated with SRD exposure. Taken together, this pattern indicates that CO shifted the myocardial inflammatory and metabolic profile toward a less injurious pattern in a marker-dependent manner, rather than producing uniform normalization across all endpoints. The attenuation of the SRD-induced increase in CB_1_R and CB_2_R expression should not necessarily be interpreted as evidence of a direct action of CO on these receptors. The present study evaluated receptor protein abundance but not their functional activation and did not include selective pharmacological tools or genetic models. Therefore, it cannot be determined whether the prevention of the increase in CB_1_R and CB_2_R observed results from a direct action of the oil on these receptors or occurs indirectly as a consequence of the overall attenuation of the metabolic, redox, and inflammatory alterations induced by the SRD. Neither possibility can be excluded based on the experimental design used. Further studies are therefore warranted to determine whether the changes observed in CB_1_R and CB_2_R are secondary to the overall effects of the oil or whether these receptors directly contribute to its cardiac effects.

Thus, these findings are consistent with CO attenuating multiple interconnected components of the myocardial response to SRD, including lipid overload, oxidative stress, inflammatory signaling, and extracellular matrix remodeling. Because the intervention consisted of a full-spectrum extract, the specific contribution of CBD, THC, minor cannabinoids, or terpenes cannot be dissected from the present design; nevertheless, the use of a defined-ratio formulation may be biologically relevant, since phytochemical interactions can yield effects that differ from those of isolated compounds. This is particularly relevant because most available studies on cannabinoids and cardiac injury have focused on isolated compounds, such as CBD or THC, often administered through routes or in models that differ substantially from nutritionally induced MetS (77) Therefore, the present study provides novel preclinical evidence that an orally administered full-spectrum cannabis formulation can prevent early myocardial responses to dietary metabolic overload.

Previous studies support the biological plausibility of these findings. In diabetic and hypertensive models, cannabinoid-based interventions — particularly isolated CBD or medicinal CO — have been reported to reduce myocardial ROS production, lipid peroxidation, inflammatory signaling, adhesion molecule expression, and fibrosis, while improving antioxidant defenses and cardiac injury markers (78–82). Moreover, CB2-oriented interventions have been associated with reduced oxidative stress, improved antioxidant responses, and attenuation of fibrosis through mechanisms involving Nrf2 and TGF-β signaling (34). In line with this mechanistic framework, SRD-fed rats in the present study exhibited cardiac Nrf2 downregulation together with TGF-β upregulation, suggesting that these pathways may participate in the early redox and profibrotic response induced by sugar overload. Importantly, although these studies differ from ours in disease model, cannabinoid composition, route, dose, and treatment duration, they collectively support the capacity of phytocannabinoid-based interventions to modulate redox-inflammatory and remodeling pathways relevant to cardiac injury.

Several limitations should be acknowledged. First, the involvement of the ECS should be regarded as associative rather than causal. Endocannabinoid levels, the expression and activity of their metabolic enzymes, receptor-specific functional activation, and the cellular source of the CB_1_R/CB_2_R signal were not assessed, the latter because receptors were quantified in whole-tissue homogenates. Second, because CO was administered as a full-spectrum extract—without antagonists, isolated cannabinoids, or receptor-deficient models—its effects cannot be ascribed to a specific constituent or receptor pathway; this, however, reflects the deliberate translational focus of the study on the whole formulation. Third, the present study was conducted exclusively in male rats. Although our group has also performed studies in female rats using the same SRD model and the same CO formulation (51), those studies focused on metabolic, hemodynamic, and hepatic parameters and therefore do not allow us to determine whether the cardiac effects observed in the present study are reproduced in females. Given that sex-related differences may exist in cardiovascular physiology, metabolism, and regulation of the endocannabinoid system, future studies should specifically evaluate these effects in female animals. In addition, the present study did not include experiments designed to determine whether the observed changes in CB_1_R and CB_2_R expression play a functional role in the cardiac alterations or in the effects of CO. Therefore, these findings should be interpreted as associations, and further studies will be required to determine the potential involvement of these receptors.

Furthermore, future studies applying cannabis-based interventions in a therapeutic setting —administered once the cardiometabolic response is established, and over longer exposure periods— will help clarify whether the preserved CB_1_R/CB_2_R expression reflects a direct receptor-level action or an indirect consequence of preventing the underlying metabolic and inflammatory event.

Overall, these results identify the heart as an early target of sucrose-driven metabolic overload and indicate that even short-term SRD feeding is sufficient to induce a coordinated profile of lipotoxicity, oxidative stress, inflammation, endothelial activation, and CB_1_R/CB_2_R expression remodeling in the absence of detectable electrocardiographic differences at this point in time. Within this preventive experimental context, concomitant daily oral administration of full-spectrum CO prevented or attenuated multiple components of this early cardiometabolic response. Given the limited availability of comparable preclinical studies using orally administered full-spectrum cannabis extracts in nutritional models of MetS, our findings provide novel evidence supporting the potential of phytocannabinoid-based interventions as modulators of early myocardial responses associated with metabolic overload.

## 5 Conclusion

The present study demonstrates that short-term exposure to a sucrose-rich diet is sufficient to induce early cardiac remodeling, characterized by coordinated metabolic, redox-inflammatory, and structural alterations, together with changes in cardiac cannabinoid receptor expression, despite the absence of overt electrocardiographic abnormalities. These findings identify the heart as an early and dynamic target of diet-induced metabolic stress. The concomitant administration of a full-spectrum CO with a CBD:THC 2:1 ratio prevented or attenuated multiple features of this early cardiac response, supporting a preventive effect against the development of diet-induced cardiometabolic dysfunction. Taken together, these findings contribute to understanding the early cardiac response to diet-induced metabolic overload and support further investigation of the preventive potential of cannabis-derived formulations against the development of cardiometabolic alterations.

## Data Availability

The original contributions presented in the study are included in the article/supplementary material, further inquiries can be directed to the corresponding author.

## Glossary

ACC: acetyl-CoA carboxylase
CAT: catalase
CB_1_R: cannabinoid receptor type 1
CB_2_R: cannabinoid receptor type 2
CBD: cannabidiol
CO: cannabis oil
CK-MB: creatine kinase - muscle-brain
CPT-1: carnitine palmitoyltransferase-1
dBP: diastolic blood pressure
ECG: electrocardiogram
ECS: endocannabinoid system
FAS: fatty acid synthase
F4/80: macrophage marker F4/80
G6PDH: glucose-6-phosphate dehydrogenase
GPx: glutathione peroxidase
GR: glutathione reductase
GSH: reduced glutathione
H&E: hematoxylin and eosin
4-HNE: 4-hydroxynonenal
IHC: immunohistochemistry
iNOS: inducible nitric oxide synthase
IOD: integrated optical density
LDH: lactate dehydrogenase
MCP-1: monocyte chemoattractant protein-1
ME: malic enzyme
MetS: Metabolic Syndrome
NEFA: non-esterified fatty acids
NF-κB: nuclear factor kappa B
Nrf2: nuclear factor erythroid 2-related factor 2
PAI-1: plasminogen activator inhibitor-1
PPARα: peroxisome proliferator-activated receptor alpha
QTc: corrected QT interval
RD: Reference Diet
ROS: reactive oxygen species
sBP: systolic blood pressure
SRD: sucrose-rich diet
SRD + Ca: SRD + CO
SREBP-1c: sterol regulatory element-binding protein 1c
TBARS: thiobarbituric acid reactive substances
TEM: transmission electron microscopy
TG: triglyceride
TGF-β: transforming growth factor-β1
THC: tetrahydrocannabinol
TNF-α: tumor necrosis factor-alpha
VCAM-1: vascular cell adhesion molecule-1
